# Oxidative Stress, Glutathione Insufficiency, and Inflammatory Pathways in Type 2 Diabetes Mellitus: Implications for Therapeutic Interventions

**DOI:** 10.3390/biomedicines13010018

**Published:** 2024-12-26

**Authors:** John Dawi, Yura Misakyan, Stephen Affa, Samuel Kades, Ananya Narasimhan, Fouad Hajjar, Max Besser, Kevin Tumanyan, Vishwanath Venketaraman

**Affiliations:** 1 College of Osteopathic Medicine of the Pacific, Western University of Health Sciences, Pomona, CA 91766, USA; john.dawi@westernu.edu (J.D.); yura.misakyan@westernu.edu (Y.M.); samuel.kades@westernu.edu (S.K.); ananya.narasimhan@westernu.edu (A.N.); fouad.hajjar@westernu.edu (F.H.); max.besser@westernu.edu (M.B.); 2 Department of Chemistry, Physics, and Engineering, Los Angeles Valley College, Valley Glen, CA 91401, USA; affasn7875@student.laccd.edu; 3 College of Podiatric Medicine, Western University of Health Sciences, Pomona, CA 91766, USA; kevin.tumanyan@westernu.edu

**Keywords:** T2DM, GSH, ROS, Vitamin D3, IL-6, β-cells

## Abstract

Type 2 diabetes mellitus (T2DM) is significantly associated with oxidative stress, resulting from the imbalance between reactive oxygen species (ROS) production and antioxidant defenses. This imbalance contributes to insulin resistance, β-cell dysfunction, and complications in organs like the vasculature and nervous system. Glutathione (GSH), a major antioxidant, is crucial for neutralizing ROS, but GSH levels are notably low in T2DM, exacerbating oxidative stress and inflammation. Elevated interleukin-6 (IL-6) levels further intensify inflammation and oxidative stress, disrupting insulin signaling and worsening complications such as nephropathy, retinopathy, and neuropathy. While lifestyle modifications and antioxidant supplementation are current approaches for managing oxidative stress, their effectiveness in preventing complications remains under study. Recent investigations suggest that GSH and Vitamin D3 supplementation may offer dual-action benefits, as Vitamin D3 not only has anti-inflammatory properties but also promotes GSH synthesis. This dual action helps mitigate both oxidative stress and inflammation, addressing key pathological features of T2DM. This review highlights the complex interactions between oxidative stress, GSH insufficiency, and IL-6, and emphasizes the potential of targeted therapies to improve the management and outcomes of T2DM.

## 1. Introduction

**Figure 1:**
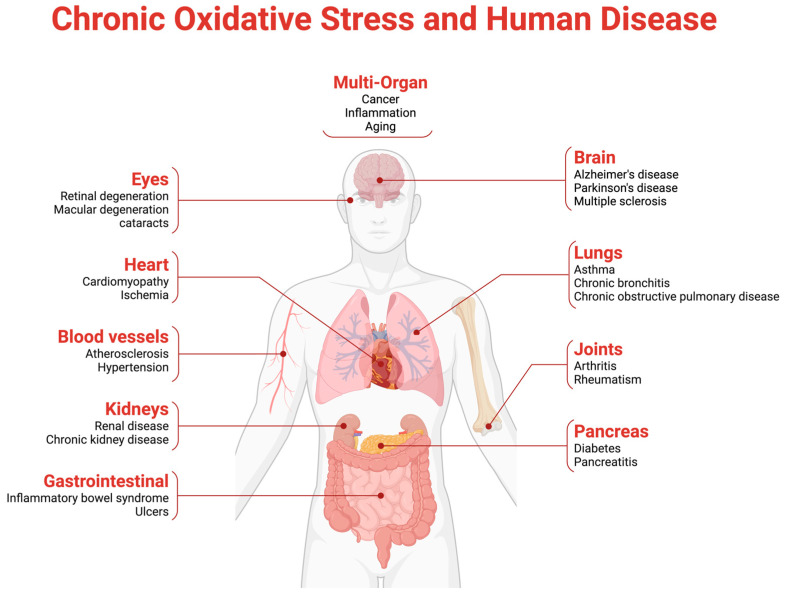
Effects of Oxidative Stress on the Human Body. Chronic oxidative stress, an imbalance of free radicals and defenses, leads to cell damage across multiple organs. In the eyes, it is linked to retinal and macular degeneration and cataracts. Cardiovascular impacts include cardiomyopathy, ischemia, atherosclerosis, and hypertension, while the kidneys are affected by renal disease and chronic kidney disease. In the gastrointestinal tract, it contributes to inflammatory bowel syndrome and ulcers. In the brain, oxidative stress is associated with Alzheimer’s, Parkinson’s, and multiple sclerosis; in the lungs, it is tied to asthma, bronchitis, and COPD. It also impacts the joints, causing arthritis and rheumatism, and the pancreas, relating to diabetes and pancreatitis. Broadly, oxidative stress accelerates aging, drives inflammation, and contributes to cancer and other chronic diseases across the body.

Oxidative stress is a major contributor to the pathogenesis of type 2 diabetes mellitus (T2DM) and its associated complications, resulting from an imbalance between reactive oxygen species (ROS) and the body’s antioxidant defenses [1]. While ROS are natural byproducts of cellular metabolism, they increase significantly under chronic hyperglycemia, exacerbating oxidative stress [2]. Hyperglycemia drives oxidative stress through several pathways, including the polyol and hexosamine pathways and the formation of advanced glycation end products (AGEs) [3]. These pathways contribute to cellular damage by producing ROS that overwhelm antioxidant systems [2]. The oxidative stress linked to hyperglycemia impairs insulin signaling and β-cell function, contributing to insulin resistance, a hallmark of T2DM [4]. This disruption of glucose homeostasis creates a self-perpetuating cycle of oxidative damage [3]. Moreover, oxidative stress is closely linked to diabetic complications like retinopathy, nephropathy, and cardiovascular diseases [1] (Figure 1).

Specifically, oxidative stress exacerbates endothelial dysfunction by damaging the vascular endothelium, causing ROS overproduction and AGE formation that impair vasodilation and promote chronic inflammation [2]. Current therapies target oxidative stress through antioxidants, lifestyle modifications, and dietary changes, although the efficacy of these approaches in preventing diabetic complications is still being studied [3]. Exploring oxidative stress mechanisms in diabetes offers insights into new therapeutic options to manage T2DM and its complications [1].

**Figure 2:**
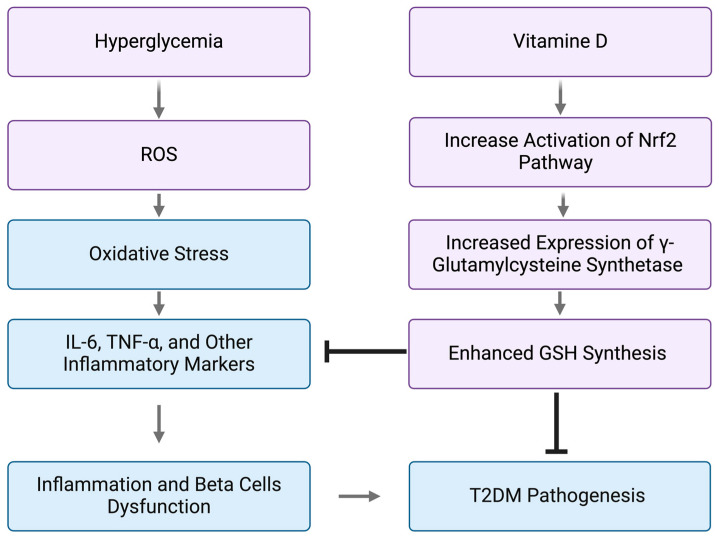
Role of Vitamin D on GSH Synthesis. GSH reduces ROS and oxidative stress. Reduced oxidative stress reduces the progression of T2DM.

Glutathione (GSH) is a vital intracellular antioxidant that protects cells from oxidative damage, particularly relevant to diabetes where GSH depletion is implicated in insulin resistance and β-cell dysfunction (Figure 2). Decreased GSH levels are consistently seen in T2DM patients, reflecting oxidative stress worsened by hyperglycemia [4,5]. T2DM patients often have reduced GSH synthesis due to increased demand from oxidative stress, complicating the condition further [6,7]. GSH is also crucial for maintaining cellular redox balance, but in diabetes, ROS overwhelms antioxidant defenses, causing cellular damage and progression of complications [4,5]. Restoring GSH levels could improve insulin signaling and glucose metabolism while reducing oxidative stress [4,5]. The association between GSH levels and glycemic control underscores its therapeutic potential in diabetes management [6,7]. GSH also plays a role in detoxifying harmful metabolites and regulating immune responses, highlighting its importance in managing diabetes-related inflammation and tissue damage [5,7]. Research suggests that replenishing GSH could be a promising therapeutic strategy to address diabetes complications, warranting further investigation [4,5].

Interleukin-6 (IL-6) is a pro-inflammatory cytokine critical in diabetes pathophysiology, primarily through its roles in inflammation and insulin resistance. Elevated IL-6 levels in obesity are linked to T2DM development, underscoring IL-6’s role in metabolic dysregulation [8,9]. IL-6 disrupts insulin signaling by activating pathways that phosphorylate insulin receptor substrates (IRS), leading to reduced insulin receptor activity and insulin resistance, a hallmark of T2DM. IL-6 also activates suppressor of cytokine signaling (SOCS) proteins, which inhibit insulin signaling and glucose uptake, worsening hyperglycemia [10]. Chronic inflammation marked by elevated IL-6 disrupts systemic metabolism, activating other pro-inflammatory cytokines like TNF-α and IL-1, creating a feedback loop that intensifies inflammation and insulin resistance [11,12]. IL-6 further contributes to the liver’s acute-phase response by promoting C-reactive protein (CRP) synthesis, an inflammation marker linked to T2DM risk and poor glycemic control [8,13]. Targeting IL-6 with receptor antagonists shows potential for reducing inflammation and improving glycemic control in T2DM patients, with promising clinical trial results [14]. Additionally, lifestyle changes such as diet and exercise can reduce IL-6 levels, presenting non-pharmacological options for managing diabetes-related inflammation [15]. Given IL-6’s role in diabetes pathophysiology, more research into its molecular mechanisms could lead to novel approaches for preventing T2DM complications.

Exploring IL-6-induced oxidative stress related to GSH insufficiency is crucial in understanding T2DM’s multifactorial nature. Elevated IL-6 levels promote ROS and inflammatory markers, leading to cellular damage and complications like nephropathy, retinopathy, and cardiovascular diseases. GSH’s antioxidant role neutralizes ROS, but inadequate GSH exacerbates oxidative stress, leading to apoptosis and dysfunction in various tissues, worsening diabetic complications [16]. This interaction between IL-6 and GSH insufficiency disrupts insulin signaling, promoting insulin resistance and impaired glucose metabolism [16,17]. Understanding these interactions is essential for developing targeted therapies to mitigate the impact of oxidative stress on diabetic complications.

Given the harm of oxidative stress and GSH insufficiency, investigating GSH and Vitamin D3 supplementation as potential therapies is compelling. GSH supplementation may bolster antioxidant defenses, alleviate diabetes-associated oxidative stress, and improve glycemic control [18]. Meanwhile, Vitamin D3, with its anti-inflammatory properties, can reduce IL-6 levels and enhance insulin sensitivity, suggesting its utility as an adjunct therapy in T2DM management [19]. Both GSH and Vitamin D3 offer protective effects against oxidative stress, and Vitamin D3’s role in GSH synthesis further enhances its potential against diabetes complications [18,19]. Exploring these supplements could lead to innovative strategies for reducing oxidative stress and improving outcomes for T2DM patients. Continued research is essential to validate effective interventions that enhance quality of life for those affected by T2DM.

## 2. Oxidative Stress in Diabetes

Oxidative stress, defined as an imbalance between reactive oxygen species production and antioxidant defense, is central to the pathophysiology of diabetes and its complications. In diabetes, high glucose levels stimulate ROS production through various pathways, including the mitochondrial electron transport chain, nicotinamide adenine dinucleotide phosphate (NADPH) oxidase, and advanced glycation end products (AGEs). Mitochondrial dysfunction, particularly in hyperglycemic conditions, results in excess ROS due to impaired electron transfer, which promotes superoxide production [20]. NADPH oxidase, activated by hyperglycemia, generates ROS in vascular and immune cells, contributing to endothelial dysfunction and inflammatory signaling [21]. AGEs, which form through non-enzymatic reactions between glucose and proteins, engage receptors like RAGE, activating nuclear factor kappa B (*NF-κB*) pathways and further promoting ROS production and inflammatory cytokine release [22]. These sources lead to a sustained oxidative environment that exacerbates insulin resistance, β-cell dysfunction, and vascular damage, characteristic of diabetes pathology [23].

ROS have been implicated in several diabetic complications, including nephropathy, neuropathy, and retinopathy. Through direct oxidative damage to lipids, proteins, and DNA, ROS impair cellular function and survival in tissues prone to diabetic damage, particularly the kidneys, nerves, and retinal cells [24]. Oxidative stress also activates inflammation via upregulation of *NF-κB*, which promotes the transcription of inflammatory mediators, perpetuating a cycle of inflammation and ROS generation [25]. Chronic oxidative stress and inflammation contribute to the failure of antioxidant defenses in diabetic patients, highlighting the importance of balancing ROS and antioxidants as a therapeutic strategy [26].

Interleukin-6 is a pleiotropic cytokine with a dual role in diabetes and oxidative stress. Produced by adipose tissue, endothelial cells, and immune cells in response to hyperglycemia, IL-6 levels are elevated in diabetic patients, where they play a role in insulin resistance and chronic inflammation (1). IL-6 signaling is mediated by two main pathways: classic signaling through membrane-bound IL-6 receptors (IL-6R) on immune cells, and trans-signaling through soluble IL-6R, which can activate cells lacking IL-6R via glycoprotein 130 (gp130) [27]. Classic signaling tends to promote immune homeostasis, while trans-signaling has been implicated in inflammatory processes and is associated with metabolic disorders [28]. Through trans-signaling, IL-6 activates the Janus kinase-signal transducer and activator of transcription (JAK-STAT) pathway, enhancing *NF-κB* and leading to increased production of ROS and pro-inflammatory cytokines [29].

In diabetes, elevated IL-6 exacerbates oxidative stress by upregulating NADPH oxidase activity and ROS production, particularly in adipose and muscle tissues, contributing to systemic inflammation and insulin resistance [30]. IL-6-mediated oxidative stress further impairs insulin signaling pathways, aggravating glucose intolerance [31]. Additionally, IL-6 stimulates other pro-inflammatory cytokines such as TNF-α and IL-1β, perpetuating a cycle of inflammation and oxidative stress that damages vascular and pancreatic β-cells [32]. By linking inflammation, oxidative stress, and insulin resistance, IL-6 plays a central role in diabetic pathology, making it a potential target for therapeutic interventions aiming to reduce ROS and inflammation in diabetic patients [33].

## 3. GSH Insufficiency and Diabetic Complications

Glutathione (GSH), a tripeptide composed of glutamine, cysteine, and glycine, serves as a key antioxidant that supports cellular detoxification and protection. As a primary defense against oxidative stress, GSH neutralizes reactive oxygen species and sustains redox homeostasis. It functions via two main mechanisms: xenobiotic detoxification through conjugation, and hydrogen peroxide (H₂O₂) reduction, facilitated by the enzyme glutathione peroxidase. During this reduction, GSH is converted to glutathione disulfide (GSSG) and is subsequently regenerated by glutathione reductase, thus maintaining cellular redox equilibrium. This cycle is vital in protecting cellular structures, such as lipid membranes, proteins, and DNA, from oxidative damage, emphasizing the importance of GSH in cellular survival and function [34].

### 3.1. GSH Insufficiency and Diabetes: Impact on Pancreatic β-Cell Function and Insulin Resistance

Type 2 diabetes mellitus (T2DM) is characterized by elevated oxidative stress and reduced GSH levels, which detrimentally affect pancreatic β-cell function and insulin sensitivity. Due to their low antioxidant enzyme expression, β-cells are especially vulnerable to oxidative damage. GSH depletion exacerbates ROS buildup, leading to oxidative damage, impaired insulin secretion, and eventual β-cell apoptosis. Research shows that oxidative stress-induced β-cell dysfunction significantly contributes to diabetes progression [35].

Additionally, GSH insufficiency is linked to insulin resistance. ROS accumulation disrupts insulin signaling by inhibiting insulin receptor substrate (IRS) proteins, which subsequently reduces the translocation of glucose transporter type 4 (GLUT4) to the plasma membrane and lowers glucose uptake in peripheral tissues such as muscle and adipose tissue. Chronic oxidative stress also promotes the release of pro-inflammatory cytokines like tumor necrosis factor-alpha (TNF-α), which further exacerbates insulin resistance [36]. Thus, GSH is crucial for both β-cell function and insulin sensitivity, with its depletion representing a significant factor in diabetes pathogenesis.

### 3.2. Diabetic Complications: Vascular Damage and Neuropathy

Oxidative stress is central to the development of diabetic complications, particularly those affecting the vascular system and nervous tissue. Vascular endothelial cells are highly susceptible to ROS, and GSH depletion heightens oxidative stress within the vasculature. Endothelial dysfunction—marked by reduced nitric oxide (NO) bioavailability, impaired vasodilation, and increased vascular permeability—is a primary feature in both microvascular and macrovascular diabetic complications [25]. GSH protects endothelial cells by neutralizing ROS and sustaining NO levels.

GSH insufficiency accelerates the formation of advanced glycation end products (AGEs), which bind to receptors on endothelial cells and trigger pro-inflammatory and pro-thrombotic responses that contribute to vascular damage [37]. This is particularly evident in conditions like diabetic retinopathy and nephropathy, where oxidative stress-induced microvascular damage leads to retinal and kidney dysfunction, respectively.

Diabetic neuropathy is similarly associated with oxidative stress and GSH depletion. ROS-induced damage to nerve cells and impaired blood flow to peripheral nerves are linked to insufficient GSH levels. Restoration of GSH, either through supplementation or enhanced endogenous production, has demonstrated efficacy in reducing oxidative damage in animal models of diabetic neuropathy, underscoring GSH’s role in protecting neural tissue [38].

### 3.3. Oxidative Stress, GSH Insufficiency, and Endothelial Dysfunction in Diabetes

Endothelial dysfunction in diabetes largely stems from oxidative stress, with GSH depletion playing a critical role. ROS inhibit endothelial nitric oxide synthase (eNOS), reducing NO production and compromising vasodilation. This endothelial dysfunction contributes to the onset and advancement of diabetic vascular complications, such as coronary artery disease (macrovascular) and retinopathy (microvascular) [39].

In endothelial cells, GSH insufficiency promotes oxidative stress, increasing the expression of adhesion molecules like vascular cell adhesion molecule-1 (VCAM-1) and intercellular adhesion molecule-1 (ICAM-1). These molecules facilitate leukocyte adherence to the endothelium, which promotes inflammation and contributes to atherosclerosis. Consequently, GSH insufficiency-induced endothelial dysfunction is implicated in both microvascular complications, like diabetic nephropathy and retinopathy, and macrovascular complications, such as myocardial infarction and stroke [24].

### 3.4. Oxidative Stress, GSH Insufficiency, and Vascular Complications in Diabetes

Research highlights the link between oxidative stress, GSH insufficiency, and diabetic complications. For example, Johansen et al. 2005, found that T2DM patients had significantly lower GSH levels than healthy controls, correlating with higher oxidative stress markers and vascular dysfunction [40]. This study suggests that GSH depletion contributes to the heightened cardiovascular risk in diabetic patients.

Further studies, such as Maritim et al. 2003, have reviewed the role of oxidative stress role in diabetic complications, finding that GSH supplementation improves endothelial function and lowers vascular complications in diabetic animal models [41]. Additionally, GSH precursors like N-acetylcysteine (NAC) have shown promise in enhancing endogenous GSH synthesis and minimizing oxidative damage in diabetes.

In clinical settings, GSH levels are often inversely correlated with HbA1c levels, indicating that GSH depletion worsens as glycemic control deteriorates, increasing complication risks. Interventions targeting oxidative stress and GSH insufficiency through diet, pharmacology, or antioxidant therapies could potentially reduce the burden of vascular complications in diabetic patients.

GSH is essential for cellular detoxification and protection against oxidative stress. GSH insufficiency in diabetes contributes to β-cell dysfunction, insulin resistance, and the development of complications. In diabetic patients, oxidative stress and GSH depletion promote endothelial dysfunction, accelerating both microvascular and macrovascular damage. Therapeutic approaches aimed at restoring GSH levels hold potential for mitigating diabetic complications, highlighting the significance of oxidative stress management in diabetes care.

## 4. Neuropathy and Nerve Damage in Diabetes

Diabetic neuropathy, a common and debilitating complication of diabetes, stems largely from hyperglycemia-induced oxidative stress, which increases reactive oxygen species production and damages peripheral nerves [42]. This nerve injury manifests as pain, tingling, and a progressive loss of sensation in extremities [43]. A critical antioxidant, glutathione (GSH), neutralizes ROS and provides protection against oxidative damage; however, GSH levels are often low in diabetic patients, exacerbating nerve damage and accelerating neuropathy progression [44]. Though efforts to restore GSH and other antioxidant pathways have been explored, clinical applications have been limited by individual variability and other confounding factors [45]. Mitochondrial dysfunction, intricately linked to oxidative stress, further contributes to neuropathic damage in diabetic patients [46]. Research on mitochondrial-targeted therapies, such as those aimed at maintaining mitochondrial integrity and function, is ongoing, but the majority of these therapies are in early experimental phases with few clinical trials substantiating their effectiveness [47]. Protecting or restoring mitochondrial function represents a critical avenue for future diabetic neuropathy therapies, as the loss of mitochondrial efficiency directly correlates with nerve cell degeneration [48].

Moreover, mitochondrial dysfunction, characterized by disrupted dynamics, reduced membrane potential, and impaired oxidative phosphorylation, exacerbates neuropathy in diabetes. Research highlights that these mitochondrial changes are driven by hyperglycemia-induced ROS, leading to bioenergetic deficits in neuronal cells. Studies in animal models, such as streptozotocin (STZ)-induced diabetic rodents, demonstrate that mitochondrial-targeting antioxidants like mitoquinone (MitoQ) can partially restore mitochondrial function and alleviate neuropathic symptoms [49]. In hyperglycemia, excess glucose overwhelms several metabolic pathways, including the polyol and hexosamine pathways, generating harmful byproducts and ROS that damage neurons. Biomarkers like nerve growth factor (NGF), brain-derived neurotrophic factor (BDNF), and oxidative stress markers (e.g., malondialdehyde) are gaining attention for their utility in early neuropathy detection. Preclinical studies have shown that these markers correlate with neuronal degeneration, supporting their translational value in human studies [50]. Advanced imaging modalities like corneal confocal microscopy (CCM) are also being explored to detect early nerve fiber loss in diabetic patients.

Promising treatments for neuropathy include antioxidants like alpha-lipoic acid (ALA) and anti-inflammatory agents targeting neuroinflammation pathways. Clinical trials, such as the NATHAN I study, demonstrated that ALA significantly improved neuropathy symptoms over four years. In preclinical studies, combined therapies using mitochondrial-targeting molecules and ROS scavengers showed synergistic effects in protecting nerve fibers [51]. These findings highlight the potential of integrating preclinical insights into clinical strategies for neuropathy management

**Figure 3:**
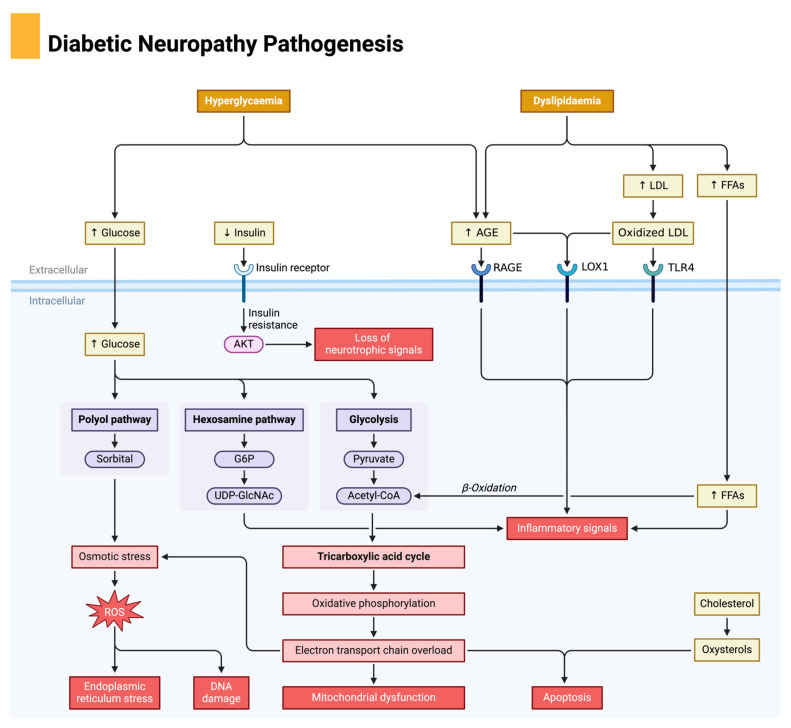
Pathophysiology of diabetic neuropathy. Diabetic neuropathy is driven by high blood sugar (hyperglycemia) and elevated blood fats (dyslipidemia). Excess glucose enters cells, causing insulin resistance and activating pathways that generate harmful byproducts. These byproducts, such as sorbitol and advanced glycation end products (AGEs), lead to oxidative stress, DNA damage, and osmotic imbalances, which strain cellular functions. Meanwhile, dyslipidemia introduces oxidized LDL, which binds to receptors and triggers inflammation. Both hyperglycemia and dyslipidemia overload the mitochondria—the cell’s energy centers—causing an imbalance in energy production and more oxidative stress. The combined effects of oxidative stress, inflammation, and mitochondrial dysfunction damage nerve cells, ultimately leading to their death and the development of diabetic neuropathy.

Diabetic neuropathy results from a combination of high blood sugar (hyperglycemia) and high blood fats (dyslipidemia). In hyperglycemia, excess glucose enters cells and overwhelms several metabolic pathways, including the polyol, hexosamine, and glycolysis pathways [48]. This leads to the production of harmful byproducts, osmotic stress, and reactive oxygen species, causing oxidative stress, DNA damage, and endoplasmic reticulum stress. Insulin resistance also develops, reducing the nerve-supporting effects of insulin [48]. Simultaneously, dyslipidemia increases LDL cholesterol and fatty acids, leading to the formation of advanced glycation end-products (AGEs) and oxidized LDL. These molecules activate specific cell receptors (RAGE, LOX1, and TLR4), which trigger inflammatory responses and further contribute to cell damage. The excess fatty acids and glucose-derived metabolites also overload the mitochondria, disrupting their function and leading to more ROS production and oxidative stress. All these stressors together create an inflammatory environment and disrupt mitochondrial function, ultimately leading to apoptosis (cell death) in nerve cells [48]. This cascade of metabolic disturbances and cellular stress causes the nerve damage characteristic of diabetic neuropathy, which leads to pain, numbness, and loss of sensation in affected individuals. (Figure 3).

In clinical settings, neuropathy is often diagnosed at an advanced stage due to the limitations of current diagnostic methods [52]. Enhanced biomarkers and advanced imaging modalities are under investigation to improve early detection, allowing for more timely interventions that could slow or prevent progression [53]. Earlier diagnosis may enable broader treatment options that would mitigate the risk of severe neuropathy and improve overall outcomes for diabetic patients [46].

Although promising, much of the current research on diabetic neuropathy treatments is limited to smaller trials, with a need for broader studies across diverse populations. Age, sex, comorbidities, and lifestyle factors significantly impact neuropathy progression, underscoring the need for tailored, patient-specific approaches to therapy [52]. Larger studies that examine the roles of these variables are essential to refining personalized treatment approaches, which may ultimately offer more effective management strategies for diabetic neuropathy [44].

### Immune Dysfunction and Comorbidities in Diabetes

Oxidative stress in diabetes also impairs immune function by fostering chronic inflammation and dysregulation [47]. Elevated pro-inflammatory cytokines, such as interleukin-6, play a significant role in driving persistent inflammation in diabetic patients, leading to weakened immunity and impaired wound healing, which increases susceptibility to infections and related complications, such as diabetic foot ulcers [43]. These ulcers exemplify the combined effect of oxidative stress and chronic inflammation, where inflammation and ROS together create a feedback loop that exacerbates immune dysfunction and worsens neuropathy, complicating patient care [42,46].

Modulating inflammatory pathways, particularly IL-6, presents a promising therapeutic target for managing both neuropathy and immune dysfunction. Reducing IL-6 activity has shown potential in alleviating inflammation and improving immune responses, though safely targeting these pathways without compromising immunity remains challenging [48,52]. Further research is needed to establish the effectiveness and safety of such anti-inflammatory therapies in diabetic populations [44]. Emerging research supports dual-action therapies that combine antioxidants with anti-inflammatory agents, aiming to simultaneously reduce oxidative stress and control inflammation [53]. Although largely theoretical, these combination therapies could address the underlying causes of both neuropathy and immune dysfunction. Early studies indicate that targeting both pathways may offer a comprehensive strategy to improve patient outcomes, warranting additional investigation in clinical trials [45]. Recent studies strongly support dual-action therapies combining antioxidants and anti-inflammatory agents as promising approaches to managing diabetes-related immune dysfunction and comorbidities. These therapies simultaneously address oxidative stress and chronic inflammation, both central to the pathology of diabetes and its complications.

Notably, curcumin, resveratrol, and cinnamon have demonstrated efficacy in reducing markers of oxidative stress, such as malondialdehyde (MDA), and inflammatory mediators like interleukin-6 (IL-6) and tumor necrosis factor-alpha (TNF-α). These phytochemicals enhance total antioxidant capacity (TAC) and positively regulate carbohydrate and lipid metabolism. For instance, curcumin has shown potential in decreasing glycated hemoglobin and low-density lipoprotein (LDL) levels while increasing high-density lipoprotein (HDL), improving overall metabolic control in diabetes [54]. Another study indicated that a combination of Vitamin D and dipeptidyl peptidase-4 (DPP-4) inhibitors preserved β-cell function and prolonged disease remission phases in type 1 diabetes, demonstrating synergistic anti-inflammatory and immune-regulatory effects [55]. Furthermore, preclinical evidence suggests that combining DPP-4 inhibitors with histone deacetylase inhibitors or melatonin can ameliorate oxidative stress and β-cell dysfunction. These combinations show promise in improving glucose tolerance and insulin levels in animal models, reinforcing their potential as part of therapeutic regimens [55]. Additionally, a systematic review emphasized the role of antioxidant therapies in mitigating reactive oxygen species (ROS) levels, reducing cellular damage, and modulating inflammation across multiple chronic conditions, including diabetes [56].

While these studies highlight the potential of dual-action therapies, larger-scale clinical trials are necessary to confirm their safety and efficacy in diabetic populations. Integrating these approaches may offer a more comprehensive strategy for managing the multifaceted pathology of diabetes

As understanding of oxidative stress, inflammation, and immune dysfunction in diabetes deepens, personalized treatment strategies tailored to individual oxidative and inflammatory profiles may emerge as a new standard of care. Rather than focusing solely on glycemic control, future treatments could involve a more holistic approach, targeting specific metabolic, immune, and inflammatory markers unique to each patient, potentially slowing neuropathy progression and improving immune-related outcomes [52]. This personalized approach could transform diabetic care, improving quality of life and reducing complications for diabetic patients [44].

## 5. GSH and Vitamin D3 Supplementation GSH Supplementation

### 5.1. GSH Supplementation

Extensive research on glutathione (GSH), a water-soluble antioxidant, has elucidated its role in cellular homeostasis and free radical elimination, with emerging research highlighting its function in diabetes. GSH is reciprocally related to glycemic control as changes in hemoglobin A1c (HbA1c) quickly alter GSH stores. As type 2 diabetes mellitus (T2DM) is notably correlated with a decrease in GSH, supplementation may therefore assist in reducing hyperglycemia in diabetic patients [57] (Table 1).

Furthermore, systemic depletion of GSH may lower the body’s ability to address oxidative stress, especially in pancreatic β-cells which lack both extensive antioxidant capacity and capabilities to repair damaged DNA [58]. Exogenous GSH supplementation for diabetic individuals may therefore be a potential protective therapy for susceptible β-cells. Recent studies have gone even further to show restoration of β-cell functionality in animal models by blunting the widespread oxidative damage associated with diabetes [59].

Recent clinical trials suggest GSH supplementation in patients with diabetes mellitus may markedly improve their disease state and highlights its potential as an adjunctive therapy. A 6-month trial of oral GSH supplementation in diabetic patients yielded encouraging results, notably an increase in fasting insulin and a reduction in both HbA1c and oxidative damage. The decrease in HbA1c and increase in insulin secretions were most significant in the elderly subgroup over the age of 55, suggesting those patient populations may benefit most from supplementation [57]. Additional research from Madathil et al., 2023, showed a similar demographic trend with older patients responding more profoundly to the GSH supplementation [60]. A 3-week trial of oral GSH supplementation in obese males, both with and without concurrent T2DM, showed a significant increase in whole body insulin sensitivity without altering the markers of oxidative stress. The GSH supplemented group experienced a ~19% increase in skeletal muscle GSH, without a concurrent increase in their mitochondrial hydrogen peroxide emission rate [61].

Contrasting the above studies, an investigation to restore depleted GSH in adolescents with type 1 diabetes mellitus (T1DM) with supplementation was unable to replicate the alleged benefits of GSH supplementation. The 3-month study failed to replenish the patients’ GSH pools or alleviate their markers of oxidative stress [62].

Glutathione (GSH), a water-soluble antioxidant, plays a critical role in maintaining cellular homeostasis and mitigating oxidative stress. Extensive research shows that GSH levels inversely correlate with HbA1c, highlighting its therapeutic potential in diabetes management. However, pharmacokinetic studies indicate that oral GSH supplementation has variable absorption rates and limited bioavailability. Alternative methods, such as intravenous GSH or precursors like N-acetylcysteine (NAC), enhance systemic GSH levels more effectively. In clinical trials, NAC supplementation reduced markers of oxidative stress by 25% and improved glycemic control, with HbA1c levels decreasing by 1.2% after six months [63]. While these results are promising, long-term safety profiles require further validation. Preclinical models using diabetic rodents also underscore the potential of GSH precursors in restoring redox balance and reducing β-cell apoptosis [64].

In addition to its antioxidant properties, GSH supports detoxification pathways critical for pancreatic β-cell survival. Its depletion under hyperglycemia exacerbates ROS accumulation, impairing insulin secretion. Emerging dual-action therapies combining GSH with anti-inflammatory agents have shown promise in preclinical studies. For example, combining NAC with curcumin significantly improved insulin sensitivity and reduced inflammatory markers like TNF-α in diabetic models [65].

### 5.2. Vitamin D3 Supplementation

Alongside GSH, Vitamin D has also emerged as a potential therapeutic supplement for diabetes management. While previous studies have shown conflicting results regarding the usefulness of Vitamin D supplementation, a recent meta-analysis review from Musazadeh et al., 2023, has yielded favorable results. Researchers concluded that supplementation in diabetic patients demonstrated improved insulin sensitivity, increased glucose absorption, decreased HbA1c and decreased fasting blood sugar. It was noted that these benefits may especially decrease diabetic-associated complications such as vascular diseases. The most significant effects of supplementation were realized within the first 15 weeks as dosages below 4000 IU. One mechanism attributed to these outcomes is believed to be the stimulation of β-cell L-type calcium channels, thereby increasing insulin secretions [66]. Another mechanism may be a reduction of inflammatory mediators; one study demonstrated suppression of TNF-α and CRP in diabetic patients taking Vitamin D supplements [67]. Furthermore, Vitamin D may act as an antioxidant by suppressing the oxidative stress generated by hyperglycemia in diabetic patients [68]. Vitamin D3 has emerged as a potential adjunctive therapy in diabetes management. It not only improves glycemic control but also combats oxidative stress (Table 1).

The exact mechanism involves suppression of NADPH oxidase activity, reducing ROS generation. Vitamin D3 also activates *nuclear factor erythroid 2–related factor 2 (Nrf2*), a key transcription factor that regulates antioxidant response elements. In a study by Zhao et al., 2022, diabetic patients receiving 4000 IU/day of Vitamin D3 showed a 35% reduction in inflammatory markers like IL-6 and TNF-α, alongside a 10% improvement in insulin sensitivity [65]. These effects were more pronounced in patients with baseline Vitamin D deficiency, underscoring the need for personalized dosing strategies. Preclinical studies confirm that Vitamin D3 upregulates GSH synthesis by enhancing the expression of γ-glutamylcysteine synthetase, a rate-limiting enzyme in GSH biosynthesis [69].

### 5.3. GSH and Vitamin D3 in Preventing Diabetes Complications

GSH may provide a host of protective effects against complications associated with diabetes, especially through the theorized blunting of oxidative stress on pancreatic β- cells. Supplementation is speculated to improve β-cell function, reduce HbA1c, increase insulin secretions and sensitivity, and thereby better control hyperglycemia-related complications [57]. Cardiovascular diseases represent one of the major complications in the diabetic population as the leading cause of death. Both oxidative stress and hyperglycemia are well known causes of vascular damage. Recent studies have highlighted the role of both GSH and Vitamin D in modulating the oxidative stress of diabetes, the later through stimulating GSH plasma levels [70]. Other research has suggested Vitamin D may provide benefits through increasing insulin sensitivity and the resulting increase in glucose absorption [62]. Through the aforementioned mechanisms, both Vitamin D and GSH may be strongly indicated supplements for diabetic patients, in an effort to reduce the disease burden and lower future complications.

Glutathione (GSH) and Vitamin D3 play crucial roles in combating oxidative stress, a significant contributor to the complications of diabetes. GSH, a critical intracellular antioxidant, lowers oxidative stress by neutralizing reactive oxygen species (ROS) and restoring the antioxidant capacity of enzymes like glutathione peroxidase. This regulation prevents ROS-induced damage to cellular structures, such as lipids, proteins, and DNA, which is pivotal in mitigating diabetes complications like nephropathy and neuropathy. Furthermore, GSH impacts mitochondrial function, reducing oxidative damage and improving glucose metabolism in diabetes [16,71]. Vitamin D3 contributes to oxidative stress reduction through multiple mechanisms. It inhibits the thioredoxin-interacting protein (TXNIP), a key regulator of ROS production, which in turn downregulates the NLRP3 inflammasome, reducing inflammation and oxidative stress-induced tissue damage. This pathway is especially relevant in diabetic nephropathy, where Vitamin D3 protects renal tissues from fibrosis and apoptosis. Additionally, Vitamin D3 influences pancreatic β-cell function by modulating calcium homeostasis, thereby enhancing insulin secretion and glucose regulation [16,72].

These synergistic roles of GSH and Vitamin D3 underscore their therapeutic potential in addressing oxidative stress-related pathways in diabetes management. Further studies could explore their combined effects for more comprehensive intervention strategies.

## 6. Conclusions

This review highlights oxidative stress and GSH insufficiency as critical contributors to T2DM pathophysiology and complications, including neuropathy, vascular damage, and immune dysfunction. Complications such as insulin resistance and microvascular diseases, like retinopathy and nephropathy, are strongly associated with oxidative damage and inflammation. GSH depletion exacerbates these conditions by impairing redox balance and mitochondrial function. Future research should focus on personalized treatment strategies targeting oxidative stress and inflammation pathways, integrating antioxidants like GSH and Vitamin D3 with emerging therapies such as mitochondrial-targeting molecules. Dual-action therapies combining antioxidants with anti-inflammatory agents warrant further clinical investigation to determine their efficacy in reducing T2DM complications. Recent trials, such as those combining GSH precursors with IL-6 inhibitors, offer hope for improved patient outcomes [73].

## Figures and Tables

**Table 1:** Summary of Supplementation.

Supplement	Use in Diabetes Management	Dose
Glutathione (GSH)	-Enhances glycemic control by reducing HbA1c levels. -Improves insulin secretion, particularly in older adults. -Protects β-cells from oxidative stress and damage. -Increases insulin sensitivity in obese patients with/without T2DM.	-6-month oral supplementation (specific dose not provided). -3-week trial in obese males (specific dose not provided).
Vitamin D3	-Improves insulin sensitivity and glucose absorption. -Reduces HbA1c and fasting blood sugar. -Lowers diabetic complications, particularly vascular diseases. -Reduces inflammation and oxidative stress.	-Up to 4000 IU for initial 15 weeks.

## Data Availability

Data available in references.
